# Retraction: Applicability of Moorrees, Fanning, and Hunt Method for Age Estimation in Gandhinagar, India: An Orthopantomogram (OPG)-Based Retrospective Study

**DOI:** 10.7759/cureus.r183

**Published:** 2025-05-29

**Authors:** Megha C Patel, Foram Patel, Shubham S Srivastav, Disha Makwani, Chhaya Patel, Miral Mehta, Chandni J Adalja, Khushi Rathod

**Affiliations:** 1 Department of Pediatric and Preventive Dentistry, Karnavati School of Dentistry, Karnavati University, Gandhinagar, IND; 2 Department of Periodontology, Ganga Dental Clinic, Vadodara, IND; 3 Dentistry, Government Dental College and Hospital, Ahmedabad, IND

This article has been retracted by the Editors-in-Chief due to the reproduction of figures and one table without permission from a previously published work that is protected by copyright. Given that these figures and table are fundamental to the article, the integrity of the work is significantly compromised. The figures and table have been removed from the retracted article to protect copyright. The authors disagree with the decision to retract.

